# Self‐Propelled In Situ Polymerized Nanoparticles Activating the STING Pathway for Enhanced Bladder Cancer Immunotherapy

**DOI:** 10.1002/advs.202502750

**Published:** 2025-03-26

**Authors:** Lei Peng, Anguo Zhao, Rongkang Li, Yujun Liu, Daitian Tang, Dashi Deng, Qi Zhuang, Rui Liang, Shaohua Zhang, Song Wu

**Affiliations:** ^1^ Department of Urology Lanzhou University Second Hospital Lanzhou 730000 P. R. China; ^2^ Department of Urology South China Hospital Medical School Shenzhen University Shenzhen 518116 P. R. China; ^3^ Department of Urology The Fourth Affiliated Hospital of Soochow University Medical Center of Soochow University Suzhou Dushu Lake Hospital Suzhou 215000 P. R. China

**Keywords:** bladder cancer, cGAS‐STING pathway, immunotherapy, in situ polymerization, self‐propelled

## Abstract

Bladder cancer, a common malignancy of the urinary tract, presents complex therapeutic challenges, thereby necessitating the exploration of innovative treatment strategies. This study introduces a novel, self‐propelled nanomedicine delivery system that forms nanoparticles within the bladder lumen by co‐infusing dopamine hydrochloride, Mn^2+^, cGAMP, and urease into the bladder to initiate in situ polymerization. The resulting Mn‐cGAMP@PDA‐urease (DMCU) nanoparticles possess a urease‐modified surface, which acts as an engine to generate propulsive force by breaking down urea. Consequently, this process enhances nanoparticle retention in the bladder mucosa and facilitates efficient drug delivery. The self‐assembled nanoparticles activate the STING pathway, promoting dendritic cell maturation and activation of T cells, thereby enhancing anti‐tumor immune responses. These nanoparticles remain in the bladder for an extended period, significantly improving therapeutic efficacy by sustaining drug release and reducing adverse side effects. In vivo, experiments using a mouse orthotopic model of bladder cancer demonstrate that the DMCU system enhances tumor suppression and immune activation compared with conventional therapies. This novel approach integrates nanotechnology with immunomodulation to address chemotherapy resistance and improve therapeutic efficacy.

## Graphical abstract

A self‐propelled nanomedicine delivery system forms DMCU nanoparticles in the bladder via in situ polymerization of dopamine hydrochloride, Mn^2+^, cGAMP, and urease. The urease‐driven propulsion enhances retention and drug delivery. These nanoparticles activate the STING pathway, stimulating dendritic cells and T cells to boost anti‐bladder cancer immunity.

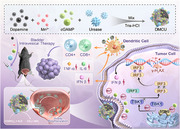

## 1 Introduction

The multifaceted challenges in cancer therapy have drawn significant attention, underscoring the need for innovative approaches to enhance therapeutic efficacy and improve clinical outcomes.^[^ ^1^, ^2^, ^3^ ^]^ Bladder cancer, a prevalent malignancy within the urinary system, presents unique opportunities for targeted intervention, attributed to the distinctive anatomical features of the bladder as a hollow organ. This structural uniqueness facilitates intravesical instillation therapy, a treatment modality that sets bladder cancer apart from other types of tumors.^[^ ^4^, ^5^ ^]^ Despite the demonstrated efficacy of intravesical chemotherapy in inhibiting tumor growth and recurrence, limitations in treatment outcomes persist, thereby highlighting the imperative for novel approaches to enhance therapeutic efficacy.^[^ ^6^, ^7^ ^]^ Recently, immunotherapy has demonstrated significant promise in treating advanced cancers; however, its effectiveness is closely tied to the immune phenotype of the tumor microenvironment (TME).^[^ ^8^, ^9^, ^10^ ^]^ Consequently, enhancing immune stimulation within the TME represents a promising strategy for treating bladder cancer.

The role of the STING pathway in tumors has been extensively studied. Free DNA acts as a critical initiator of the STING signaling pathway, which plays a pivotal role in enhancing immune responses and improving anti‐tumor therapies.^[^ ^11^, ^12^, ^13^ ^]^ Nevertheless, due to its widespread expression across various cell types, STING demonstrates dual functions, contributing to both tumor suppression and metastasis. Previous studies have demonstrated that genomic instability in tumors activates the cGAS‐STING cytosolic DNA sensing pathway, triggering downstream non‐canonical NF‐κB signaling. Furthermore, persistent chromosomal segregation errors have been demonstrated to promote cell invasion and metastasis through a STING‐dependent mechanism.^[^ ^14^ ^]^ Similarly, another study reported that the transfer of GAMR from tumor cells to astrocytes triggers the production of inflammatory cytokines and activates the signal transducer and activator of transcription 1 and NF‐κB pathways, ultimately leading to tumor growth and chemoresistance.^[^ ^15^ ^]^ Conversely, the cGAS‐STING pathway has also played a key role in advancing anti‐tumor research. Several studies have demonstrated that STING activation leads to the release of type I interferons (IFN‐I), which drive the maturation of dendritic cells (DCs). These DCs, in turn, induce and activate specific T cells, thereby enhancing cytotoxicity against tumor cells and improving drug sensitivity.^[^ ^16^, ^17^, ^18^ ^]^ Therefore, understanding the mechanisms of STING signal transduction is essential for elucidating the processes that foster anti‐tumor immunity.

In situ polymerization technology was originally developed for the synthesis of nano‐inorganic materials, which significantly improved the mechanical and functional properties of polymers.^[^ ^19^, ^20^ ^]^ However, limited medical studies have investigated the potential of in situ polymerization for delivering composite drugs in anti‐tumor therapy. The bladder is a hollow organ with distinct anatomical advantages, making it an ideal site for an intravesical in situ polymerized nano drug delivery system. This innovative therapeutic strategy combines nanotechnology with in situ polymerization, allowing drugs to be polymerized into nanoparticles directly within the bladder cavity. These nanoparticles provide numerous advantages, including efficient drug delivery, enhanced bioavailability, a stable controlled‐release system for sustained drug release, improved solubility and permeability, minimized side effects, and increased tolerance.^[^ ^21^, ^22^ ^]^ In situ polymerization further amplifies these benefits. To leverage this potential, we developed an intravesical composite drug delivery system based on in situ polymerization, offering novel insights and innovative strategies for bladder cancer treatment.

Research on micro‐ and nanorobots has resulted in substantial advancements in traditional drug delivery systems. Due to their autonomous movement and selective targeting capabilities, they enable more efficient drug delivery and prolonged drug retention times.^[^ ^23^, ^24^, ^25^ ^]^ Previous studies have explored diverse applications of nanorobots within cells, such as intracellular movement, rapid internalization‐meditated delivery, and the scavenging of reactive oxygen species.^[^ ^26^, ^27^ ^]^ Given the unique environment of the bladder as a hollow organ, urease has emerged as an efficient motor for micro‐ and nanorobots. In our previous study, we demonstrated that urease serves as the driving force for nanoparticles, facilitating self‐propulsion.^[^ ^28^ ^]^ Notably, this study is the first to integrate in situ polymerization with micro‐ and nanorobots, enhancing both propulsion and retention to achieve more efficient drug delivery, extended mucosal retention, and enhanced anti‐tumor efficacy.

Based on this context, we employed a non‐invasive intravesical instillation method to develop a novel self‐propelled, in situ polymerized nanodrug instillation system. This system enables in situ polymerization to form nanoparticles within the bladder while simultaneously modifying their surface with urease. This modification enables self‐propulsion by using urea as a fuel source, thereby enhancing drug retention and delivery efficiency. Specifically, this approach involves the co‐instillation of dopamine hydrochloride, Mn^2+^, cGAMP, and urease into the bladder within a pH 8.5 Tris–HCl buffered system. During the dopamine polymerization process, adhesive properties promote molecular adhesion and aggregation, facilitating the in situ polymerization of nanoparticles in vivo to form a novel nanoparticle, Mn‐cGAMP@PDA‐urease (DMCU).

**Figure 1:**
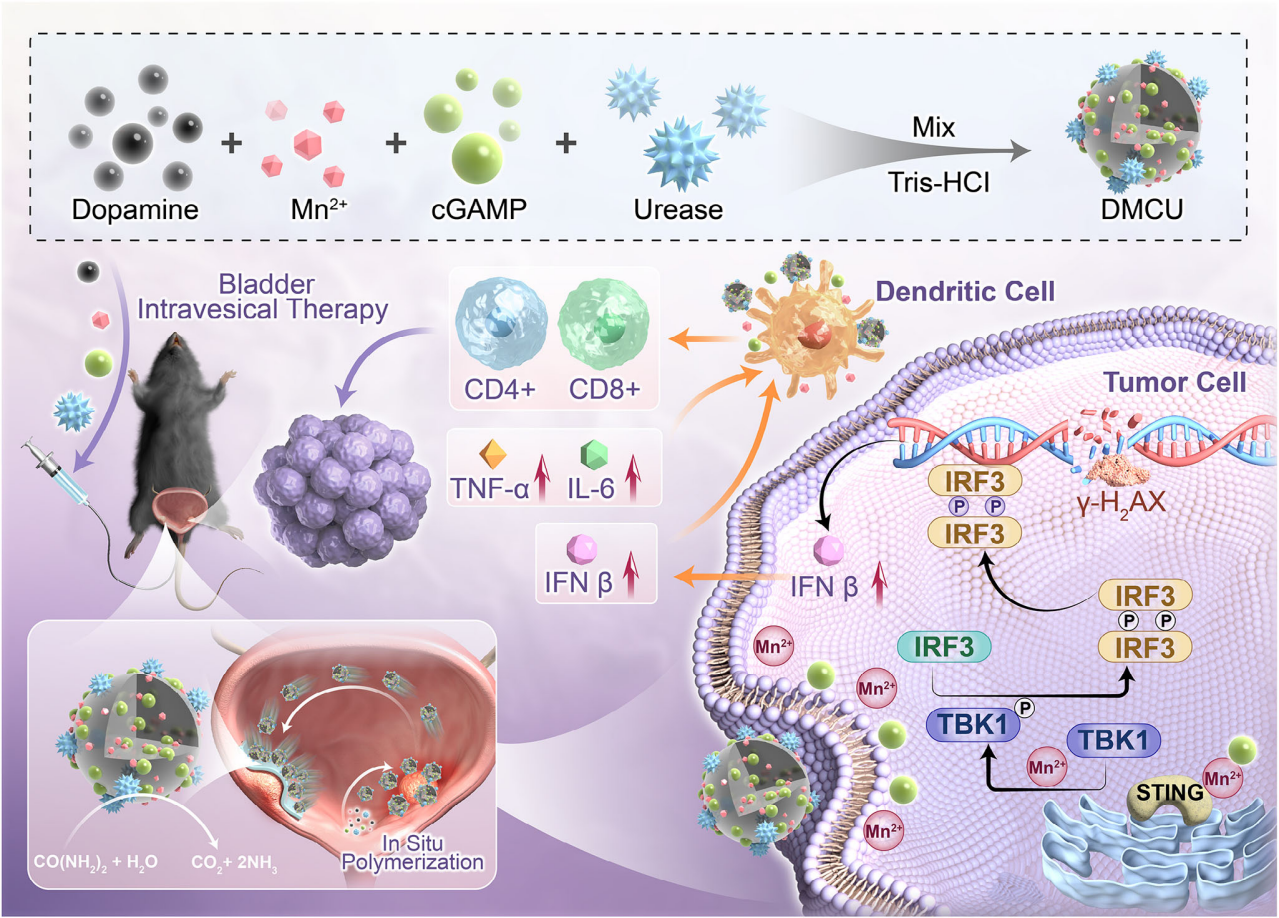
Preparation of in situ polymerized nanomedicine and schematic of the mechanism of nanoparticle formation.

The key advantage of this approach lies in its ability to enable the self‐assembly of stable nanoparticles within the bladder cavity through in situ polymerization. The urease‐modified outer shell functions as a motor, generating propulsion by breaking down urea and significantly enhancing adhesion to the bladder mucosa. A stable controlled‐release system is established within the bladder, ensuring sustained drug release and minimizing drug‐related side effects. This self‐propelled, in situ polymerized nanodrug instillation system enhances therapeutic outcomes by activating the STING pathway and promoting DCs maturation, which subsequently activates T cells, induces tumor cytotoxicity, and enhances the immunogenicity of the TME (**Figure** **1**).

## 2 Results and Discussion

### 2.1 Preparation and Characterization of DMCU

**Figure 2:**
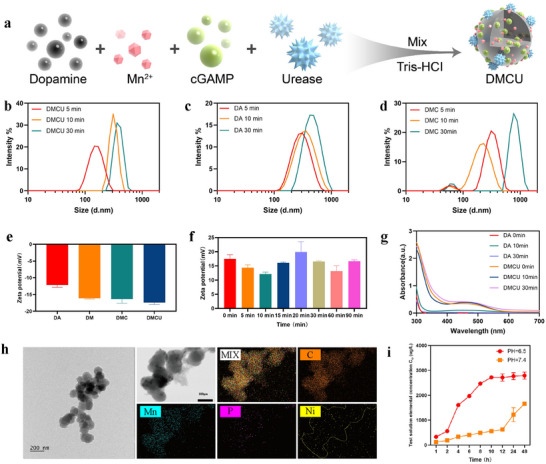
a) Schematic illustration of the polymerization process for self‐propelled, in situ polymerized nanodrugs. b–d) Particle size variations of nanomedicines DA, DMC, and DMCU at various time points before the completion of polymerization. e) Zeta potential measurements of DA, DM, DMC, and DMCU after centrifugation and purification. f) Zeta potential changes of DMCU over time in the in situ polymerization environment (Tris–HCl, pH 8.5). g) Absorption peak variations of DA and DMCU at different time points were measured using UV–vis spectroscopy. h) TEM images of DMCU and its elemental composition were analyzed via EDS mapping. Urease is a nickel‐containing oligo‐enzyme, with its active center requiring nickel ions to perform its catalytic function. Each urease molecule typically contains two nickel ions, corresponding to a mass percentage of ≈0.024%. Due to the limited amount of nickel, its location has been highlighted using a yellow dashed line for better visualization. i) Drug release from DMCU in artificial urine (pH 6.5) and PBS solution (pH 7.4) at different time points detected by inductively coupled plasma spectrometry (ICP) (where the concentration of Mn^2+^ was detected to represent the drug concentration in DMCU).

Nanoparticles were first synthesized in vitro to characterize DMCU. A one‐pot synthesis method was employed to polymerize dopamine, Mn^2+^, and cGAMP (DMC) in a pH 8.5 Tris–HCl buffered system. Urease was subsequently added, and the mixture was stirred to produce DMCU (**Figure** **2**a). Polydopamine (DA) and dopamine‐manganese ion polymers (DM) were prepared as controls using the same method. After 30 min of continuous stirring and polymerization, the mixtures were centrifuged at 14800 rpm for 20 min to collect the precipitates, which were then dispersed in 1 mL of deionized water to obtain purified nanomaterials for each group.

Dynamic light scattering (DLS) was used to measure particle size across all groups and to evaluate the physicochemical properties of the polydopamine‐based preparation system. The average particle size of the DMCU nanoparticles was ≈193 ± 27.6 nm (Figure 2b), while those of DA and DMC were 276 ± 43.5 nm (Figure 2c) and 206 nm (Figure 2d), respectively. Zeta potential measurements were conducted for each group, yielding values of −12.12 ± 0.71, −16.09 ± 0.25, −16.36 ± 1.23, and −17.39 ± 0.57 mV for DA, DM, DMC, and DMCU, respectively (Figure 2e). To evaluate the practical application of the nanodrug and the effect of in situ polymerization, the zeta potential of DMCU was measured at different time points in the pH 8.5 Tris–HCl system, revealing an average zeta potential of DMCU was 15.79 ± 2.75 mV (Figure 2f). In order to clarify the stability of DMCU nanoparticles in different solution systems, we set up an artificial urine group, a phosphate Buffered Saline (PBS) group, and a double‐distilled water (ddwater) group, and performed dynamic monitoring using DLS for 48 consecutive hours to clarify the changes in the particle size of DMCU nanoparticles. In artificial urine with pH 6.5, DMCU particle nanoparticles would continue to polymerize over time until the end of 48 h, the particle size of DMCU was 1909 ± 132.34 nm; in PBS solution with pH 7.4, the particle size of DMCU had a similar trend but the polymerization rate was slower than that in artificial urine, until the end of 48 h, the particle size of DMCU was 1076± 57.66 nm; in ddwater, the DMCU remained stable until the end of the 48 h observation time, and the particle size of DMCU was 745 ± 17 nm. This particle size trend is more in line with our concept of intravesical therapy, because DMCU has both the role of decomposition of urea and thus self‐propulsion, as well as adhesion polymerization, and in the limited intravesical therapy, the particle size of DMCU was 745 ± 17.31 nm. These abilities allow DMCUs to form a “membrane” on the bladder wall during the limited duration of intravesical therapy, thus enhancing drug delivery and retention over a long period of time (Figure S1, Supporting Information).

Due to the functional groups on the polydopamine surface, DMCU exhibits zwitterion behavior, with an isoelectric point of ≈4. At neutral pH in the purified nanodrug system, the DA group predominantly displays a negative potential. Mn^2+^, as a metal ion, effectively catalyzes dopamine polymerization, promoting the process and increasing the negative charges on the surface. Furthermore, under neutral conditions, both cGA MP and urease carry negative charges, thereby enhancing the negative potential in the purified DMC and DMCU groups. However, in practical applications, such as the intravesical instillation of nanomaterials, all components of the nanodrug are introduced into the bladder alongside the pH 8.5 Tris–HCl system. In this environment, excess Mn^2+^ remains in a free state, continuously balancing the negative charges in the system, which explains the positive zeta potential observed in DMCU under these conditions.

Ultraviolet‐visible (UV–vis) spectroscopy was used to measure the absorption peaks of DA and DMCU. The results indicate that DMCU exhibits a prominent absorption peak at ≈480 nm, similar to DA, confirming the successful synthesis of DMCU (Figure 2g). Transmission electron microscopy (TEM) was employed to further examine the morphology of the nanoparticles. As illustrated in Figure 2g and Figures S2–S4 (Supporting Information), DA, DM, DMC, and DMCU, all exhibit an irregular spherical shape and adhere to one another, verifying the adhesive properties of polydopamine. Energy‐dispersive X‐ray spectroscopy (EDS) mapping was conducted to analyze the elemental composition of DMCU, confirming the successful loading of Mn^2+^, cGAMP, and urease (Figure 2h; Figure S5, Supporting Information). In order to observe the polymerization process of DMCU and its related nanoparticle systems, we took diluted samples of DA, DM, DMC, and DMCU at 5, 10, and 30 min, respectively, and recorded their morphology under TEM. What can be seen is that the DA group polymerized at a slower rate and gradually formed adherent DA nanoparticles at the end, whereas the DM, DMC, and DMCU groups polymerized at a gradually accelerated rate, and at the end of the polymerization, all of them formed clearer nanoparticles (Figure S6, Supporting Information).

Purification centrifugation was performed at the end of polymerization in Tris‐HCl system according to the total amount of Mn^2+^ as 5 mg of feed. The total amount of Mn^2+^ in the supernatant after centrifugation was 1.17 mg, and after washing again with ddwater and centrifugation again, the total amount of Mn^2+^ in the supernatant was 0.02 mg, and the purified DMCU was degraded using aqua regia, and after that, the total amount of Mn^2+^ in the degradate clock was detected to be 3.81 mg, and the drug loading rate of DMCU could be obtained to be 76.2%. In the dialysate, the concentration of Mn^2+^ in solution was measured at different time points to determine drug release from the DMCU. From the drug release curves, it can be seen that in artificial urine, Mn^2+^ reached the peak of release in ≈10 h; while in PBS solution, the concentration of Mn^2+^ was still in the rising stage of release after 48 h (Figure 2i).

### 2.2 Self‐Propulsion Performance and Bladder Retention of DMCU

Urease is an enzyme that catalyzes the hydrolysis of urea to form ammonia and carbon dioxide. In living organisms, urease is widely found in bacteria, fungi, plants and some invertebrates. Urease specifically breaks down urea into ammonia and carbon dioxide, and the propulsive force generated by the production of carbon dioxide provides the propulsive force for nanoparticles to move:

(1) $$\left(NH_{2}\right)CO_{2}+H_{2}O\underset{\mathrm{Urease}}{\to }2NH_{3}+CO_{2}$$

**Figure 3:**
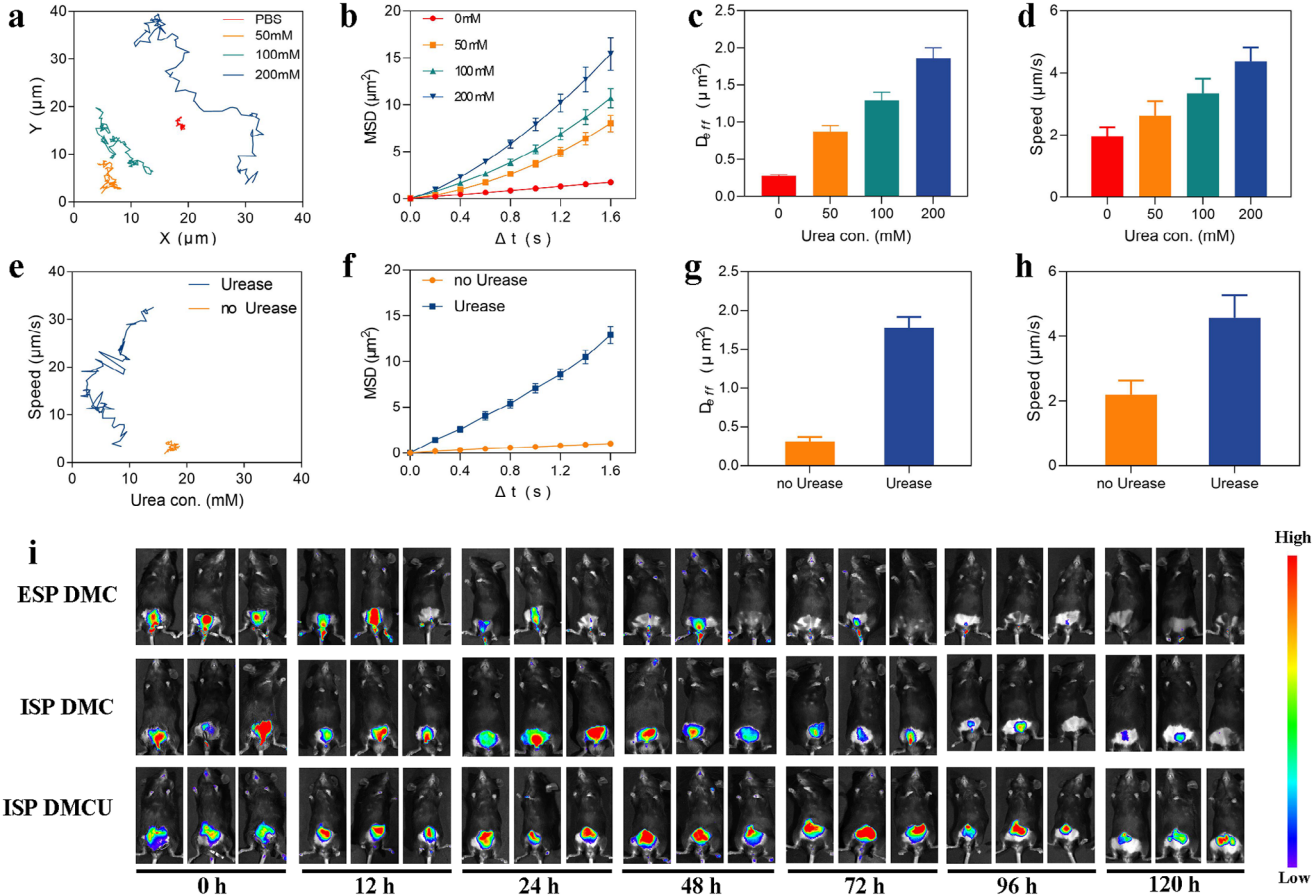
Motion performance of DMCU. a) Typical movement trajectories (over 25 s), b) MSD, c) diffusion coefficient (D*_eff_*), and d) velocities (n = 15; mean ± SEM) of the DMCU robots in PBS solutions with varying urea concentrations. e) Typical movement trajectories (over 25 s), f) MSD, g) D*_eff_*, and h) velocities of the unmodified and urease‐modified DMCUs in urine from mice with bladder tumors (n = 15; mean ± SEM). i) Exogenously polymerized (ESP) Cy5.5‐labeled DMC, in situ polymerized (ISP) DMC, and in situ polymerized DMCU drugs were administered intravesically in a mouse model of bladder cancer. The retention time of each nanoparticle in the bladder was monitored over a period of 120 h.

To confirm that urease provides active and effective propulsion for DMCU, trajectory analysis was performed in urea solutions with varying concentrations to simulate the urine environment. DMCU was placed in phosphate buffer solutions containing urea concentrations of 0, 50, 100, and 200 mm, and its movement trajectories were observed and recorded using an inverted microscope. The results indicate that the movement trajectories of DMCU extended significantly with increasing urea concentration (**Figure** **3**a), and the mean square displacement (MSD) increased linearly with time (Figure 3b), demonstrating a positive correlation with urea concentration. Additionally, the diffusion coefficient (D*_eff_*) (Figure 3c) and speed (Figure 3d) also exhibited a positive correlation with urea concentration. Representative videos of DMCU movement in PBS solutions with different urea concentrations are provided in Movies S1–S4 (Supporting Information).

Previous studies have demonstrated that the high concentration of urea in human and mouse urine provides ample fuel for urease‐loaded nanoparticles. Accordingly, sterile midstream urine was collected from mice with bladder tumors, and the urea concentration was measured at 573.0 ± 12.6 mm. The motility of unmodified DMC and DMCU in this urine sample was subsequently evaluated. The results indicate that DMCU exhibited effective movement trajectories, whereas unmodified DMC displayed only Brownian motion. Significant differences in MSD, D*_eff_*, and speed were observed between the two groups (Figure 3e–h). Representative videos of urease‐modified DMCU and non‐urease‐modified DMC in real urine are provided in Movies S5 and S6 (Supporting Information).

To further validate whether DMCU, incorporating in situ polymerization and urease as a propulsion motor, could move within a physiological environment and enhance nanoparticle adhesion to the bladder wall, exogenously polymerized DMC@Cy5.5, in situ polymerized DMC@Cy5.5, and in situ polymerized DMCU@Cy5.5 (labeled with Cy5.5 dye, red) were instilled into the bladders of three groups of mice. Fluorescence imaging was employed to evaluate the retention time of nanoparticles in the bladder across the groups. The results indicate that the in situ polymerized DMCU group (ISP‐DMCU) retained strong fluorescence signals in all three mice 120 h after instillation, whereas the fluorescence signals in the other two groups had notably weakened or disappeared (Figure 3i).

We used the same method to validate whether DMCUs combining in situ polymerization and urease as a propulsive driving force could move and enhance the mucosal penetration of nanoparticles with the bladder wall in a physiological environment. Cy5.5‐labeled Exogenously polymerized (ESP) DMCs, ISP DMCs, and ISP DMCUs were infused into the bladder of mice acting for 2 h and then observed by fluorescence imaging, and the bladder mucosal penetration in the ISP DMCU group was the ISP DMCU group had a significantly stronger penetrating effect on the bladder mucosa, and a stronger Cy5.5 fluorescence signal could still be observed in the submucosa (Figure S7, Supporting Information).

### 2.3 In Vitro Anti‐Tumor Activity and Cellular Uptake of DMCU

**Figure 4:**
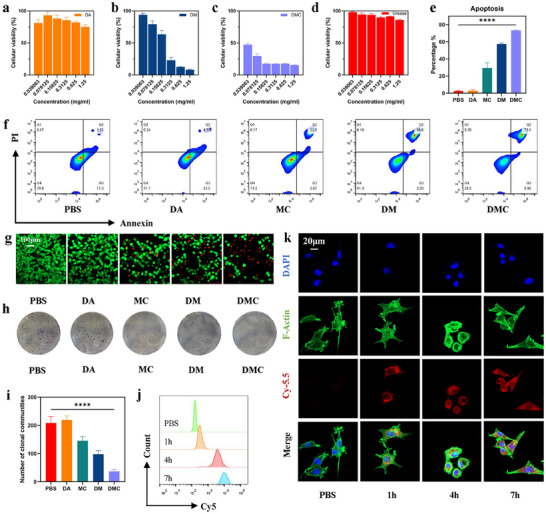
In vitro evaluation of the tumor cell uptake and cytotoxicity of DMCU. a–d) Effects of different groups of in situ polymerized nanoparticles (DA, DM, and DMC) and free urease at different concentrations on MB49 cell viability, assessed using the N‐tetramethyl‐p‐phenylenediamine method. e,f) FCM analysis graphs and semi‐quantitative evaluation of apoptosis in MB49 cells. g) CLSM images of MB49 cells stained with Calcein‐AM (green, viable) and PI (red, dead). h,i) MB49 cell colonies stained with crystal violet and semi‐quantitative analysis. j) FCM profiles demonstrating the intracellular uptake of DMC by MB49 cells at different time points. k) CLSM images of DMC@Cy5.5 co‐cultured with MB49 cells for 1, 4, and 7 h. Cell nuclei were stained with 4′,6‐diamidino‐2‐phenylindole (DAPI, blue), and actin filaments (F‐actin) were stained with phalloidin (green).

Given that urease primarily decomposes urea within the bladder to generate propulsion and exhibits minimal intrinsic cytotoxicity on its own, DMC nanoparticles were used in cell‐based experiments to investigate the in vitro anti‐tumor activity of DMCU. The cytotoxicity of DA, DM, MC, DMC, and urease against MB49 cells was measured using the resazurin assay. All groups, except for DA, were quantified based on Mn^2+^ content. The results indicate that the cytotoxicity of DMC was significantly higher than that of the other groups (**Figure** **4**a–d; Figures S8 and S9, Supporting Information). Further analysis of cell apoptosis revealed that the apoptosis rate of MB49 cells in the DMC group (≈73.1%) was markedly higher than that of cells in the other groups (Figure 4e,f).

DA, DM, MC, and DMC were applied to MB49 cells, subsequently stained with Calcein‐AM and propidium iodide (PI), to evaluate the anti‐tumor effects of DMC using confocal laser scanning microscopy (CLSM). The results demonstrated the strong cytotoxic effect of DMC (Figure 4g). Furthermore, the impact of various drug treatments on MB49 cell proliferation was examined using a colony formation assay, which demonstrated that DMC significantly inhibited the clonogenic potential of MB49 cells (Figure 4h,j). In summary, DMC exhibited pronounced in vitro anti‐tumor activity.

To elucidate the mechanism underlying the in vitro anti‐tumor activity of DMC, cellular uptake was visualized and quantified. DMC@Cy5.5 was co‐incubated with MB49 cells for 1, 4, and 7 h and observed using CLSM. After 4 h, DMC was predominantly internalized by the cells, primarily localizing in the cytoplasm (Figure 4k). Flow cytometry (FCM) was employed to analyze changes in fluorescence intensity within MB49 cells over time. The results indicate that intracellular red fluorescence steadily increased with incubation time, peaking at 7 h (Figure 4i). These findings suggest that DMCU is effectively internalized by tumor cells, thereby exerting cytotoxic effects.

### 2.4 DMC‐Induced DNA Damage and Activation of the cGAS/STING Pathway

**Figure 5:**
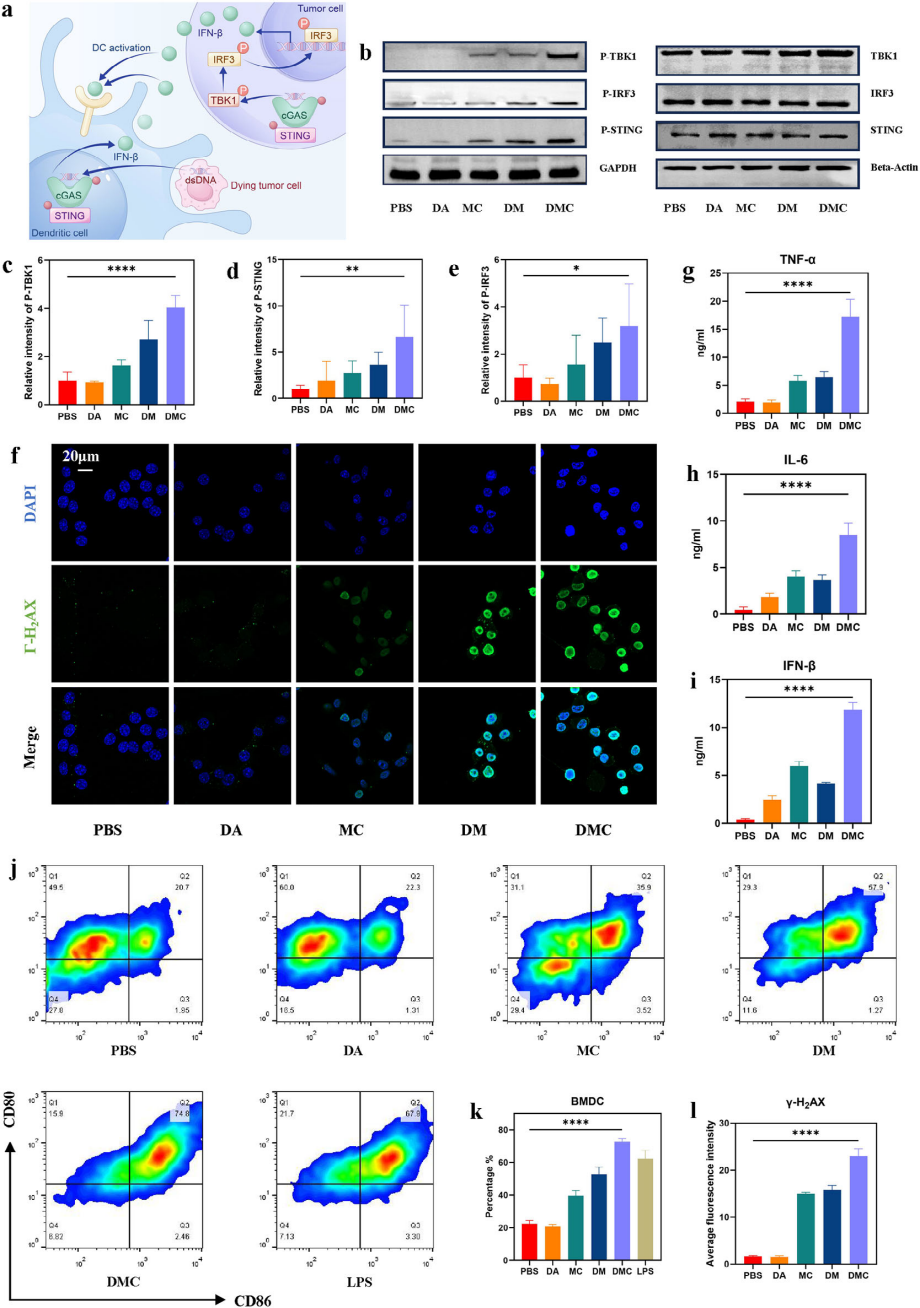
In vitro investigation of DNA damage mechanisms and STING pathway activation with DMC. a) Schematic representation of the STING pathway in tumor cells and the promotion of DC maturation. b) Western blot analysis of the STING pathway‐associated proteins, with β‐Actin and GAPDH as internal controls. c–e) Semi‐quantitative statistical analysis of STING pathway‐associated proteins Phos‐TBK1, Phos‐STING, and Phos‐IRF3 expression levels based on grayscale analysis. f) CLSM images showing γ‐H_2_AX in MB49 cells treated with different drugs (blue, DAPI; green, γ‐H_2_AX). g–i) Cytokine levels of TNF‐α, IL‐6, and IFN‐β in DC supernatants (n = 3 Petri dishes of DCs). j,k) FCM results of DC activation in co‐culture with MB49 cells treated with PBS, DA, MC, DM, and DMC. l) Semi‐quantitative statistical analysis of γ‐H_2_AX expression in MB49 cells treated with different nanomedicines.

Previous studies have shown that Mn^2+^ promotes DNA damage, leading to the release of fragmented double‐stranded DNA (dsDNA). This fragmented DNA is recognized by cGAS, which stimulates the synthesis of cGAMP. As an activator of the STING pathway, cGAMP amplifies the effects of Mn^2+^‐induced DNA damage and strengthens immune activation (**Figure** **5**a).^[^ ^29^ ^]^ The STING pathway is universally expressed in cells, and previous research has shown that nanomaterials can activate the STING pathway in tumor cells to stimulate DC maturation and induce IFN‐I production. Furthermore, certain nanomaterials, STING pathway agonists, and tumor autoantigens can also stimulate DCs, triggering cytokine production and initiating a cascade effect to enhance DC activation.^[^ ^16^, ^30^, ^31^ ^]^

To evaluate the DNA damage capability of DMC, immunolabeling of the DNA damage marker γ‐H_2_AX was performed on MB49 cells treated with various groups, followed by examination using CLSM. The results indicate a significant increase in γ‐H_2_AX expression in the DMC group, demonstrating that DMC induced DNA damage and activated the STING pathway (Figure 5f,l). To validate the activation ability of DMC on STING pathway, MB49 cells of different groups were immunolabeled with P‐STING and then detected using CLSM. The results showed that the expression of P‐STING was significantly increased in the DMC group, which indicated that DMC effectively induced the activation of the STING pathway (Figures S10 and S11, Supporting Information). Western blot analysis was performed to evaluate the expression levels of key proteins in MB49 cells. Compared with the DA, MC, and DM treatment groups, DMC‐treated cells exhibited a marked upregulation in the expression of phosphorylated STING (Phos‐STING), IRF3 (Phos‐IRF3), and TBK1 (Phos‐TBK1). These findings confirm that cGAMP significantly enhanced the Mn^2+^‐induced activation of the STING pathway (Figure 5b–e). In contrast, the expression of histone H_2_AX did not show significant differences between the groups (Figure S12, Supporting Information).

Activation of the cGAS‐STING pathway triggers the release of inflammatory cytokines, stimulates DC maturation, and induces tumor cell apoptosis through innate immune pathways. To assess the immunostimulatory capability of DMC, DCs were extracted from mouse bone marrow (BMDCs) and co‐incubated with DA, MC, DM, and DMC. The maturation status of BMDCs was assessed through FCM analysis. The results demonstrate that the proportion of mature BMDCs was highest in the DMC group (37.6%) (Figure 5j,k). In addition, after co‐incubating each drug group with MB49 cells for 24 h, the levels of interferon‐β (IFN‐β), interleukfin‐6 (IL‐6), and tumor necrosis factor‐α (TNF‐α) in the supernatant were quantified using an enzyme‐linked immunosorbent assay (ELISA). The results indicate that cytokine levels were highest in the DMC group (Figure 5g–i). In conclusion, DMC effectively induced DNA damage activated the cGAS‐STING pathway, promoted DC maturation, and enhanced anti‐tumor immune responses.

### 2.5 Biosafety and In Vivo Efficacy of DMCU

Comprehensive physiological and biochemical assessments were conducted on mice to evaluate the biosafety of DMCU. Body weight was monitored, and key serum markers of liver and kidney function were analyzed, including aspartate aminotransferase (AST), alanine aminotransferase (ALT), blood urea nitrogen (BUN), alkaline phosphatase (ALP), lactate dehydrogenase (LDH), and creatinine (CREA) (Figure S13, Supporting Information). All markers remained within normal ranges, indicating that DMCU did not cause significant adverse effects on liver or kidney function. Furthermore, hematoxylin and eosin (H&E) staining of major organs (heart, liver, spleen, lung, and kidney) revealed no significant pathological changes compared with the control group, and the organ tissue structures remained intact (Figure S14, Supporting Information). These findings confirm that DMCU demonstrates good biosafety for in vivo applications.

**Figure 6:**
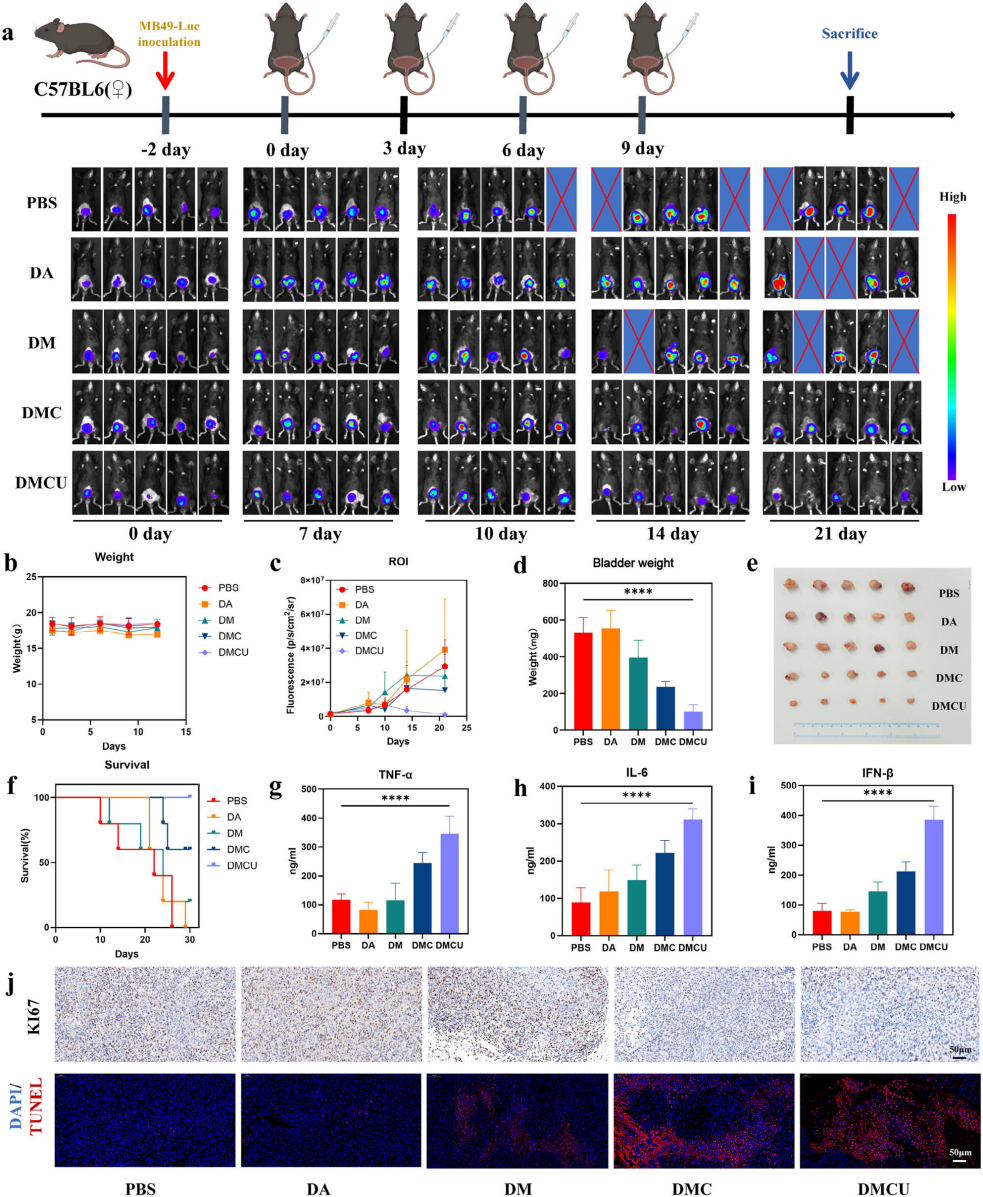
In vivo tumor growth inhibition by DMCU. a) Schematic of the treatment schedule and changes in bladder cancer fluorescence values following intravesical treatment with PBS, DA, DM, DMC, and DMCU. b) Body weight change curves of mice in each treatment group during the treatment period. c) Statistical graph showing fluorescence changes in mouse bladder tumors across treatment groups during the treatment cycle. d) Statistical graph of bladder weights in mice from each treatment group at the end of the observation cycle. e) Representative photographs of bladder tumors and f) survival curves of mice in each treatment group at the end of the observation cycle. g–i) Serum cytokines TNF‐α, IL‐6, and IFN‐β in mice treated with PBS, DA, DM, DMC, and DMCU. j) TUNEL (top) and Ki‐67 (bottom) staining of tumors extracted from mice treated with different drugs (blue: DAPI; red: TUNEL).

To evaluate the in vivo anti‐tumor efficacy of DMCU, an orthotopic bladder cancer model was established in mice using MB49‐Luciferase cells. Tumor‐bearing mice were divided into groups and treated via intravesical instillation every 3 d for a total of four treatments. After each intravesical instillation, the urethra of the mice was clamped for 1.5 h using a urethral clip to ensure adequate exposure of the drug in the bladder while avoiding drug efflux with the urethra. Changes in body weight and tumor bioluminescence imaging data were continuously monitored throughout the treatment period (**Figure** **6**a). The results indicate that, compared with the PBS group, the body weights of mice in the treatment groups fluctuated; however, no significant differences were observed (Figure 6b). Notably, the DMCU group demonstrated a marked reduction in tumor fluorescence signal after the second treatment, demonstrating its superior anti‐tumor efficacy (Figure 6c; Figure S15, Supporting Information).

Bladder tumors were harvested and weighed on day 30. The results demonstrate that the DMCU group exhibited significantly lower average bladder volumes and weight than the other groups (Since bladder tumors show infiltrative growth in the bladder, the weight and volume of the bladder are used here as a proxy for the weight and volume of the tumor) (Figure 6d,e). During the 30 d treatment and observation period, survival data were recorded for each group. Survival analysis revealed a 100% survival rate in the DMCU group, which was significantly higher than that observed in the other groups (Figure 6f). HE staining analysis of the bladders of mice at the end of the treatment (21 d) showed the smallest tumor size and the least invasiveness in the bladders of the DMCU group, validating our findings (Figures S16 and S17, Supporting Information). In addition, TUNEL and Ki‐67 staining were performed on tumor tissues. The TUNEL assay revealed a pronounced red fluorescence signal for apoptotic cells in the DMCU‐treated group (Figure 6j; Figure S18, Supporting Information). Ki‐67 immunohistochemical staining demonstrated that tumor cell proliferation activity in the DMCU group was significantly lower than in the other groups (Figure 6j). These findings further substantiate the superior in vivo anti‐tumor efficacy of DMCU. In conclusion, DMCU demonstrated excellent biosafety and remarkable anti‐tumor efficacy in mice, effectively inhibiting bladder tumor growth, prolonging survival, and reducing tumor cell proliferation.

### 2.6 In Vivo Immune Activation Effect of DMCU

**Figure 7:**
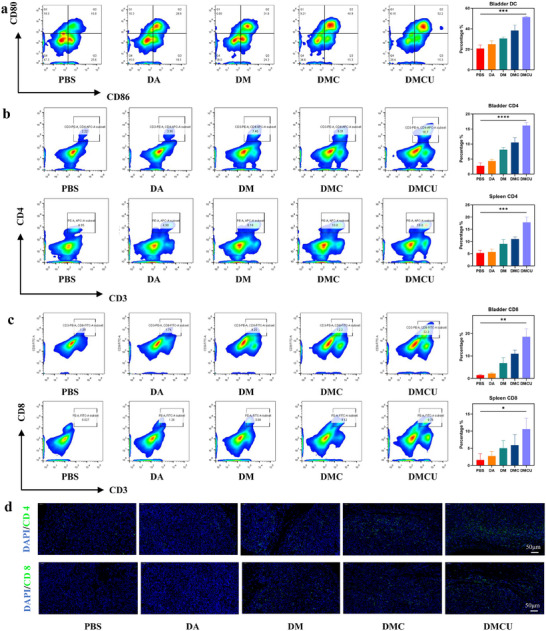
DMCU enhanced the anti‐tumor immune response through the STING pathway in vivo. a) FCM and statistical analysis of DC activation in bladder tumors of mice treated with different treatments. FCM and statistical analysis of b) CD4^+^ and c) CD8^+^ T cell activation in bladder tumors and spleens of mice treated with different drugs. e) Immunofluorescence stainingCD4 (top) and CD8 (bottom) staining of tumors extracted from mice treated with different drugs (blue, DAPI; green: CD4/CD8).

Activation of the cGAS‐STING pathway triggers the release of various cytokines, such as IFN‐β and IL‐6, which enhance anti‐tumor immune responses by recruiting immune cells to the TME and promoting DC maturation. To further assess the immune activation capability of DMCU in vivo, blood, spleen, and bladder tissues were collected from mice following bladder instillation treatment, and immune parameters were analyzed. First, serum levels of TNF‐α, IL‐6, and IFN‐β were quantified using ELISA. The results indicate that the levels of all three target cytokines in the DMCU group were significantly higher than those in the control group (Figure 6g–i). This finding confirms that DMCU activates the STING pathway and enhances immune responses, which is consistent with the in vitro results. Next, flow cytometry (FCM) was used to assess DC maturation in bladder tissue. As previously demonstrated, DMCU effectively promoted DC maturation in vitro. In the in vivo experiments, FCM analysis of bladder tumor tissues revealed that the DMCU group exhibited the highest DC maturation rate (52.2%). These data demonstrate that DMCU effectively promotes DC maturation in vivo (**Figure** **7**a).

Mature DCs activate T cells and enhance their tumor‐killing capabilities. Therefore, we further examined the activation status of CD4^+^ and CD8^+^ T cells. FCM results revealed that the proportion of CD4^+^ T cells in the spleen and bladder tissue of the DMCU group was 18.8% and 16.7%, respectively (Figure 7b). Similarly, the proportion of CD8^+^ T cells in the spleen and bladder tumor of the DMCU group was 22.3% and 8.76%, respectively (Figure 7c), both significantly higher than those observed in the other groups. Furthermore, because DMCU effectively activates immune responses, we also examined the activation of memory T cells (T_EM_) in the spleen. FCM analysis demonstrated that the activation rate of T_EM_ in the DMCU group was 26.3%, which was significantly higher than those observed in the other groups (Figure S19, Supporting Information). These results indicate that, in addition to promoting DC maturation and T cell activation in vivo, DMCU enhances immune memory responses. Finally, immunofluorescence staining was performed on the bladders of treated mice to examine the distribution of CD4^+^ and CD8^+^ T cells, revealing that the distributions of both T cell types were significantly higher in the DMCU group than in the other groups (Figure 7d).

In summary, the combination of in situ polymerization and self‐propelled particles in DMCU effectively activate the cGAS‐STING pathway, promotes DC maturation, activates CD4^+^ and CD8^+^ T cells, and significantly enhances memory T cell activity, highlighting its strong potential in tumor immunotherapy. Overall, DMCU induces a robust anti‐tumor immune response and exhibits exceptional anti‐tumor efficacy.

## 3 Conclusion

We developed a self‐propelled, in situ polymerized nanodrug system that effectively activates the STING pathway. The combination of Mn^2+^ and cGAMP significantly enhanced STING activation and amplified immune responses, thereby modulating the TME to improve tumor treatment outcomes. By delivering the nanodrug system via bladder instillation to simulate current clinical treatments, the integration of in situ polymerization and urease‐driven propulsion markedly improved nanoparticle retention and drug delivery, thereby substantially enhancing anti‐tumor efficacy. Thus, this approach offers a promising and innovative strategy for clinical tumor therapy.

## 4 Experimental Section

### Materials and Equipment

Dopamine was obtained from Merck Corporation (Darmstadt, Germany). Cyclic GMP‐AMP (cGAMP) was sourced from MedChemExpress LLC (New Jersey, USA). MnCl₂ was purchased from Aladdin (Shanghai, China). CCK‐8 was obtained from ZETA Life (San Francisco, USA). RPMI‐1640 and DMEM media, 0.25% trypsin‐EDTA, fetal bovine serum (FBS), and penicillin/streptomycin (P/S) were supplied by Gibco (Grand Island, USA). 2‐(4‐amidinophenyl)‐1H‐indole‐6‐carboxamidine (DAPI) was sourced from Shanghai Youningwei Biotechnology Co., Ltd. FITC anti‐mouse CD80, APC anti‐mouse CD86, PE anti‐mouse CD3, FITC anti‐mouse CD8, APC anti‐mouse CD4, APC anti‐mouse CD62L, and PerCP/Cyanine5.5 anti‐mouse CD44 antibodies were all purchased from BioLegend (San Diego, USA).

### Cells and Animals

MB49‐Luciferase cells were cultured in high‐glucose DMEM medium supplemented with 10% FBS and 100 U mL^−1^ penicillin‐streptomycin mixture. The cells were maintained in a humidified incubator at 37 °C with 5% CO_2_. Seven‐week‐old female C57BL/6 mice, purchased from ZhuHai Bestest Biotechnology Company (Zhuhai, China), were used in this study.

### Synthesis of DMC and DMCU

DA (5 mg), MnCl₂ (5 mg), and 2,3‐cGAMP (0.01 mg) were added to 1 mL of 10 mm Tris–HCl solution (pH 8.5) and stirred continuously at room temperature for 40 min. The mixture was then centrifuged at 14800 rpm for 20 min, and the precipitate was collected and designated as DMC. Next, 1.5 mg of urease was added and stirred for an additional 30 min at room temperature. The mixture was centrifuged again at 14800 rpm for 20 min, and the precipitate was collected and dispersed in 1 mL of deionized water, yielding 5 mg mL^−1^ of DMCU (based on Mn^2+^ concentration).

### DMCU Trajectory Tracking

The movement behavior of DMCU was assessed in PBS solutions with varying concentrations of urea (0, 50, 100, and 200 mm) or in urine collected from mice. Urea concentrations in the mouse urine were measured using an AU5821 Clinical Chemistry Analyzer (Beckman) following the protocols outlined by the manufacturer. In three independent experiments, the urea concentrations were 535.5, 512.0, and 499.3 mm, with an average concentration of 515.60 ± 18.37 mm. Videos of the movement were recorded using an inverted optical microscope (Nikon Eclipse Ti‐U) equipped with a dark‐field condenser and subsequently analyzed using NIS‐Elements AR software. The diffusion coefficient (D_eff_) was determined using the formula: MSD (Δt) = 4 D_eff_Δt, where Δt denotes the time interval.

### Synthesis of DMC@Cy5.5

DA (5 mg), MnCl₂ (5 mg), cGAMP (0.01 mg), and Cy5.5 (0.01 mg) were dissolved in 1 mL of Tris–HCl (pH 8.5) and stirred at room temperature for 30 min. The mixture was then centrifuged at 14800 rpm for 20 min, and the resulting precipitate was collected and designated as DMC@Cy5.5.

### Cellular Uptake of Nanoparticles

Coverslips were placed at the bottom of each well in a 24‐well plate, and MB49 cells (8 × 10^4^ cells/mL) were seeded into each well in 1 mL of medium and incubated at 37 °C for 12 h. The cells were treated with DMC@Cy5.5 for 1, 4, or 7 h. Following treatment, the cells were fixed with 4% paraformaldehyde, and the nuclei and cytoskeleton were stained with DAPI and Alexa Fluor 488, respectively. Images were captured using CLSM (LSM‐800, Carl Zeiss, Germany). FCM was employed to analyze the cellular uptake of the nanoparticles. MB49 cells (3 × 10^5^ cells per well) were seeded in 12‐well plates and cultured for 12 h. The cells were then treated with DMC@Cy5.5 for 1, 4, or 7 h, followed by FCM analysis (Shenzhen Weigong Bio Co., Ltd.).

### Cytotoxicity of Nanoparticles

The cytotoxicity of MB49 cells was assessed using the resazurin assay. The cells were seeded at a density of 1 × 10^4^ cells per well in 96‐well plates (Thermo Scientific, USA) and treated with DA, DM, MC, or DMC for 24 h. After removing the medium, 100 µL of a resazurin‐serum‐free medium mixture was added to each well. The plates were incubated at 37 °C for 2 h, and the absorbance at 450 nm was measured using a microplate reader (SpectraMax). All experiments were performed in triplicate. For additional analysis, MB49 cells were seeded at a density of 8 × 10^5^ cells per dish in 3.5 cm cell culture dishes (5 dishes in total). After 24 h of treatment with DA, DM, MC, or DMC, the medium was removed, and the cells were washed with cold PBS and stained with a Calcein‐AM/PI cell viability kit (Beyotime). Images were then captured using CLSM.

### Cell Apoptosis Assay

MB49 cells were seeded at a density of 3 × 10^5^ cells per well in 12‐well plates (Thermo Scientific, USA) and cultured for 12 h. After 24 h of treatment with DA, DM, MC, or DMC, the cells were harvested, stained with an Annexin V‐FITC/PI apoptosis detection kit (Beyotime), and analyzed via FCM.

### Cell Clonogenic Assay

MB49 cells were seeded in 6‐well plates at a density of 2000 cells per well and cultured for 12 h. The cells were treated with DA, DM, MC, or DMC for 10 d. Once visible colonies formed, the cells were fixed with 4% paraformaldehyde and stained with 0.5% crystal violet. Images of the colonies were then captured using a camera.

### Detection of DNA Damage Marker γ‐H_2_AX

T24 cells were seeded at a density of 1 × 10^5^ cells per well in 24‐well plates containing coverslips. After 12 h, the medium was replaced, and the cells were treated with PBS, DA, DM, MC, or DMC for 24 h. Following treatment, the cells were washed with PBS and incubated with a γ‐H_2_AX antibody at 37 °C for 2 h. Cells were then incubated with an Alexa Fluor 488‐conjugated secondary antibody for 1 h and observed using CLSM.

### In Vitro Maturation of BMDCs

BMDCs were extracted from 5‐ to 6‐week‐old female C57BL/6 mice and cultured in RPMI‐1640 medium supplemented with 10% FBS, granulocyte‐macrophage colony‐stimulating factor (20 ng mL^−1^, Peprotech), and interleukin‐4 (IL‐4) (10 ng mL^−1^, Beyotime) at 37 °C under 5% CO_2_. After 5 d of culture, pretreated MB49 cells were co‐cultured with BMDCs for 24 h. Following treatment, BMDCs were stained with anti‐CD11c‐PE, anti‐CD80‐FITC, and anti‐CD86‐APC antibodies and analyzed for DC maturation via FCM.

### Western Blot Analysis

MB49 cells were seeded at a density of 1 × 10^6^ cells per well in 6‐well plates and cultured for 12 h. After 12 h, the medium was replaced, and the cells were treated with PBS, DA, DM, MC, or DMC for 24 h. Following treatment, the cells were washed three times with cold PBS. RIPA lysis buffer containing protease and phosphatase inhibitors were added, and the proteins were extracted by centrifugation at 12000 rpm for 15 min. Protein content was measured using a BCA protein assay kit. Proteins were separated by SDS‐PAGE and transferred onto a PVDF membrane using a gel electrophoresis system (Bio‐Rad, USA). The membrane was incubated overnight at 4 °C with primary antibodies (Cell Signaling Technology, 16029T). After washing three times, the PVDF membrane was incubated with HRP‐conjugated secondary antibodies for 1 h. The membrane was then washed three times with 1× TBST and visualized using an Amersham Imager 600 (AI600, General Electric Company, USA) with 500 µL of ECL chemiluminescence substrate (Beyotime Biotechnology Co., Ltd., P0018AS).

### ELISA

ELISA kits (Shenzhen Belante Biotechnology Co., Ltd.) were prepared, and serum samples were thawed on ice. The kits were equilibrated at room temperature for 2 h. The plate was set up with standard, sample, and blank wells. Standard solution (50 µL) was added to the standard wells, sample (50 µL, in triplicate) was added to the sample wells, and nothing was added to the blank wells. Each well received 100 µL of HRP‐labeled antibody solution, and the plate was sealed with sealing film and incubated at 37 °C for 1 h. After washing five times, a 1:1 mixture of solution A and solution B was added to each well (100 µL per well), followed by incubation at 37 °C for 15 min. Subsequently, stop solution (50 µL) was added, and absorbance was measured at 450 nm using a microplate reader. Concentrations were calculated based on the standard curve.

### Establishment of Mouse Bladder Cancer Orthotopic Model

A total of 5 × 10^6^ MB49‐Luciferase cells were resuspended in 20 µL of 1× PBS. After ensuring adequate anesthesia, the mouse bladder was exposed, and the cell suspension was injected into the bladder wall. A distinct, transparent, disc‐shaped bulge was observed at the injection site upon successful injection. The abdominal cavity was then closed, and the vital signs of the mice were monitored. After 3 d, ex vivo imaging revealed fluorescence values of approximately 3 × 10^6^ p/s/cm^2^/sr.

### Tumor Growth Inhibition in MB49 Mouse Orthotopic Model

Twenty‐five mice were randomly assigned to five groups, with five mice per group. The mice were treated with the following doses via intravesical instillation: PBS, DA, DM, DMC, and DMCU (26 mg kg^−1^). Tumor fluorescence and mouse body weight measurements were recorded every 3 d. The treatment lasted for 10 d. On day 30, the mice were euthanized, and the bladders from each group were harvested, weighed, and recorded.

### Evaluation of In Vivo Immune Activation in Mice

After the treatment period, the mice were euthanized, and blood, spleen, and bladder samples were collected. Blood samples were centrifuged to isolate serum, and the levels of TNF‐α, IL‐6, and IFN‐β were measured using the corresponding ELISA kits. Spleen and bladder tissues were ground and digested, then stained with anti‐CD11c‐PE, anti‐CD80‐FITC, and anti‐CD86‐APC antibodies to evaluate DC maturation via FCM analysis. The differentiation of CD4^+^, CD8^+^, and memory T cells was also assessed.

### Ethical Approval

The study was conducted in compliance with relevant guidelines and regulations for the care and use of laboratory animals. All animal experimental procedures were approved by the Experimental Committee and Ethics Committee of South China Hospital of Shenzhen University (approval number: IACUC‐202300167).

### Statistical Analysis

Data processing and statistical analysis were performed using GraphPad Prism 9.5 software to assess the differences between groups under various experimental conditions. A t‐test was used for pairwise comparisons, while one‐way ANOVA was applied for comparisons involving three or more groups. Statistical significance was set at a *p*‐value < 0.05, with * indicating *p* < 0.05, ** representing *p* < 0.01, *** denoting *p* < 0.001, and **** indicating *p* < 0.0001. In addition, bar graphs were generated using GraphPad Prism 9.5 to visualize the experimental data.

## Conflict of Interest

The authors declare no conflict of interest.

## Author Contributions

L.P., A.Z., R.L., and Y.L. contributed equally to this work and are joint first authors. S.W., S.Z., and R.L. were responsible for the design and improvement of the project. L.P., A.Z., R.L., and Y.L. conducted all the experimental work, including the factual experiments and their replication. D.T., D.D., and Q.Z. provided guidance and optimization for the experiments. All authors reviewed the manuscript and approved its submission.

## Supporting information



Supporting Information

Supplemental Movie 1

Supplemental Movie 2

Supplemental Movie 3

Supplemental Movie 4

Supplemental Movie 5

Supplemental Movie 6

## Data Availability

The data that support the findings of this study are available from the corresponding author upon reasonable request.
