# Supplementary material for: Self‐Propelled In Situ Polymerized Nanoparticles Activating the STING Pathway for Enhanced Bladder Cancer Immunotherapy
Source: Adv Sci (Weinh). 2025 Mar 26;12(25):2502750. doi: 10.1002/advs.202502750 (PMC12224934; doi:10.1002/advs.202502750)
Supplement: Supplementary file 1 — Supporting Information [file ADVS-12-2502750-s003.docx]

**Supporting Information for**

**Self-Propelled In Situ Polymerized Nanoparticles Activating the STING Pathway for Enhanced Bladder Cancer Immunotherapy**

Lei Peng,^A,B†^ Anguo Zhao,^C†^ Rongkang Li,^A,B†^ Yujun Liu,^B†^ Daitian Tang,^B^ Dashi Deng,^B^ Qi Zhuang,^B^ Rui Liang, ^B^* Shaohua Zhang, ^B^* Song Wu^A,B^*

^A^Department of Urology, Lanzhou University Second Hospital, Lanzhou, 730000, China.

^B^Department of Urology, South China Hospital, Medical School, Shenzhen University, Shenzhen, 518116, P. R. China.

^C^Department of Urology, The Fourth Affiliated Hospital of Soochow University, Medical Center of Soochow University, Suzhou Dushu Lake Hospital, Suzhou 215000 China.

*^†^These authors contributed equally to this work and are joint first authors.*

*Corresponding authors: Song Wu, Shaohua Zhang, Rui Liang

**Email:** [wusong@szu.edu.cn](mailto:wusong@szu.edu.cn); [zhangshaohua@szu.edu.cn](mailto:zhangshaohua@szu.edu.cn); [liangrui@alu.suda.edu.cn](mailto:liangrui@alu.suda.edu.cn).

**This PDF file includes:**

Figures S1 to S19

Legends for Movies S1 to S6

**Other supporting materials for this manuscript include the following:**

Movies S1 to S6

Fig S1. Variation in particle size of DMCU post-polymerization across different solvent systems over time.


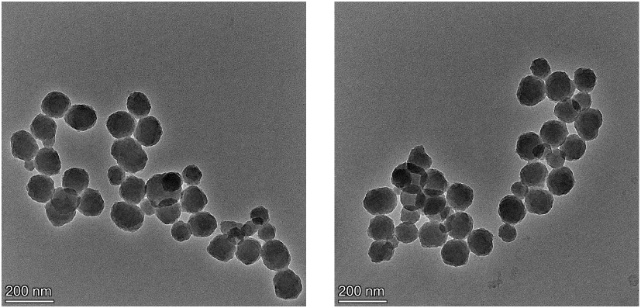


Fig. S2. Conformal characterization of PDA by transmission electron microscopy. Scale bars: 200 nm.

**
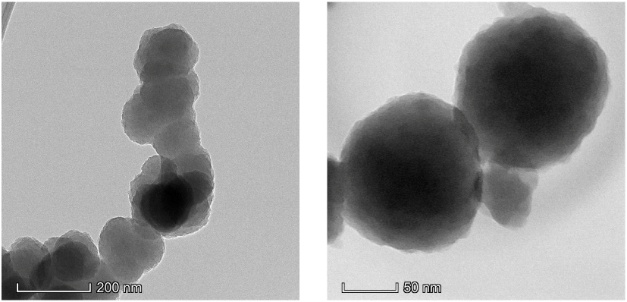
**

Fig. S3. Conformal characterization of DM by transmission electron microscopy. Scale bars: 100, 50 nm.


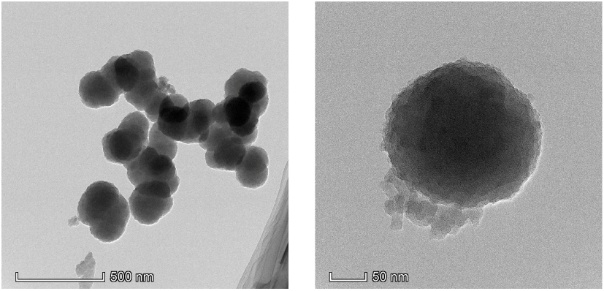


Fig. S4. Conformal characterization of DMC by transmission electron microscopy. Scale bars: 500, 50 nm.

Fig. S5. Elemental spectrograms of DMCUs were analyzed using energy dispersive X-ray spectroscopy (EDS) mapping.


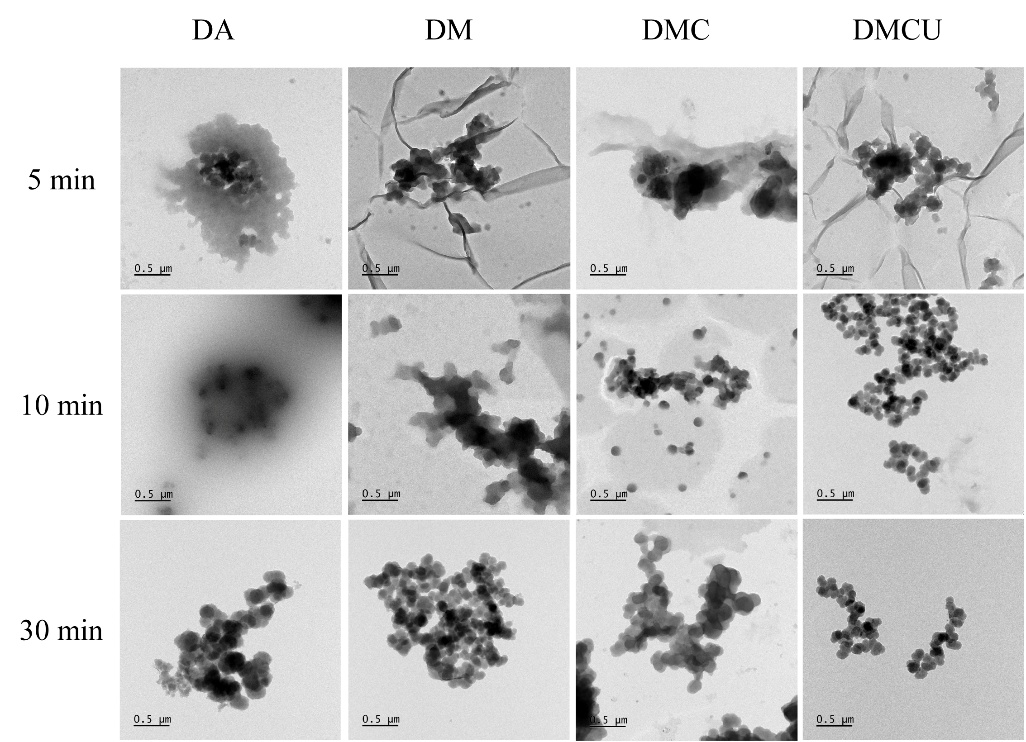


Fig. S6. Conformal characterization of DA, DM, DMC and DMCU at different time points during polymerization by transmission electron microscopy. Scale bars: 500 nm.


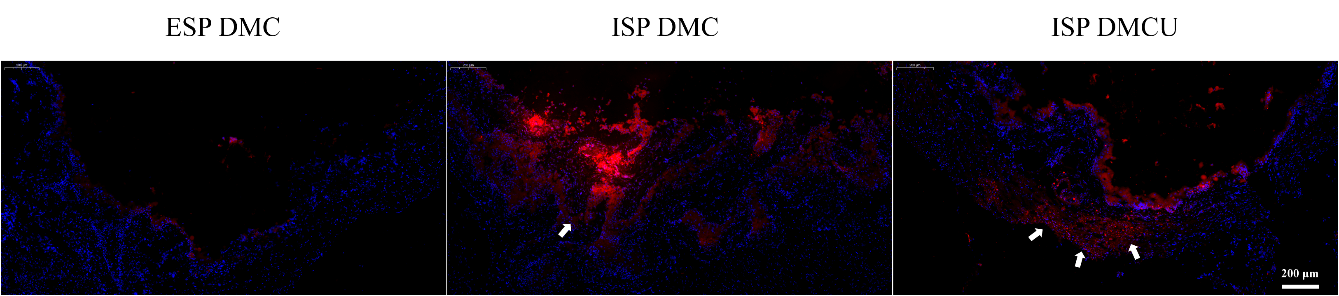


Fig. S7. Fluorescence images using cy5.5-labeled ESP DMC, ISP DMC and ISP DMCU were resolved 2 hours after perfusion into the mouse bladder; As indicated by the arrows in the ISP DMCU diagram, the DMCU penetrated into the submucosal layer of the bladder; whereas the ESP DMC penetrated only into the mucosal layer of the bladder. Scale bars: 200 μm.

Fig. S8. Statistical graph of the effect of MC group on the viability of MB49 cells at different concentrations (The concentration of Mn^2+^ was used as the unit of measurement).

Fig. S9. Statistical graph of the effect of DMC group on the viability of MB49 cells at different concentrations (The concentration of Mn^2+^ was used as the unit of measurement).


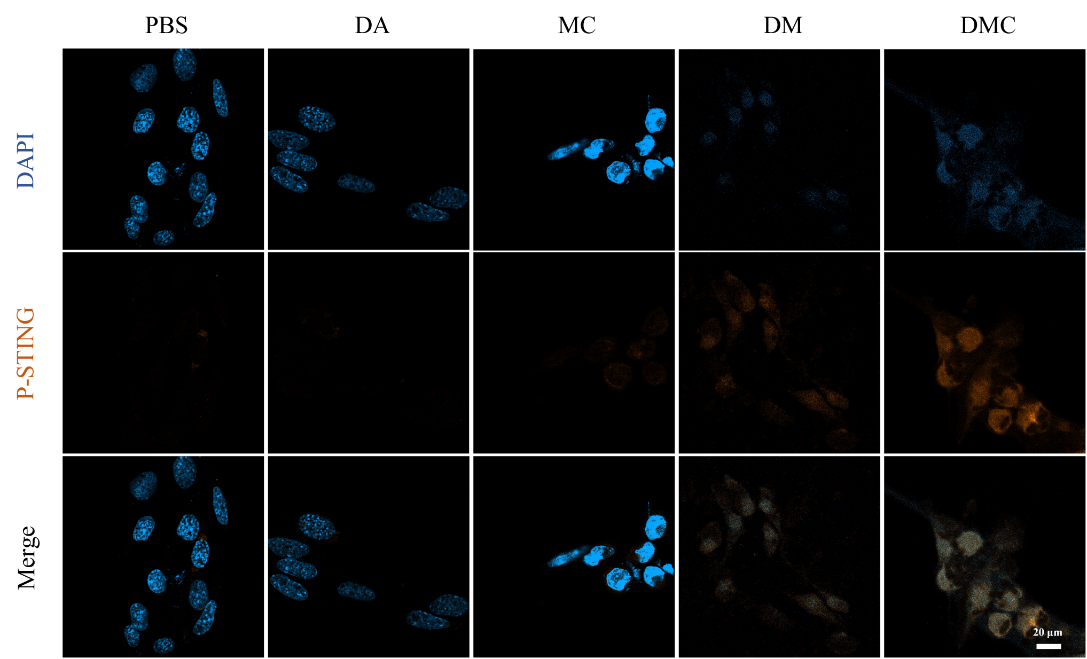


Fig. S10. CLSM images showing P-STING in MB49 cells treated with different drugs (blue, DAPI; yellow, P-STING). (Dopamine hydrochloride turns black upon oxidative polymerization, resulting in less than bright DAPI staining in the DA, DM, and DMC groups.) Scale bars: 20μm.

Fig. S11. Statistical analysis of the mean fluorescence intensity of CLSM images of P-STING.


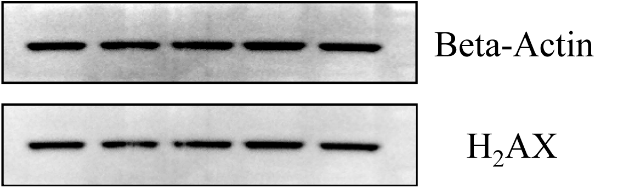


Fig. S12. Western blot analysis of the H_2_AX proteins, with β-Actin as internal controls.

Fig. S13. Serum hepatic and renal function indices of mice treated with different nanomedicine groups were analyzed for: a) total bilirubin (TBIL), b) direct bilirubin (DBIL), c) aspartate aminotransferase (AST), d) alanine aminotransferase (ALT), e) total bile acids (TBA), f) albumin (ALB), g) γ-glutamyl aminotransferase (γ-GT), h) Alkaline phosphatase (ALP), i) Creatinine (CREA), j) Urea nitrogen, k) and Uric acid (UA).


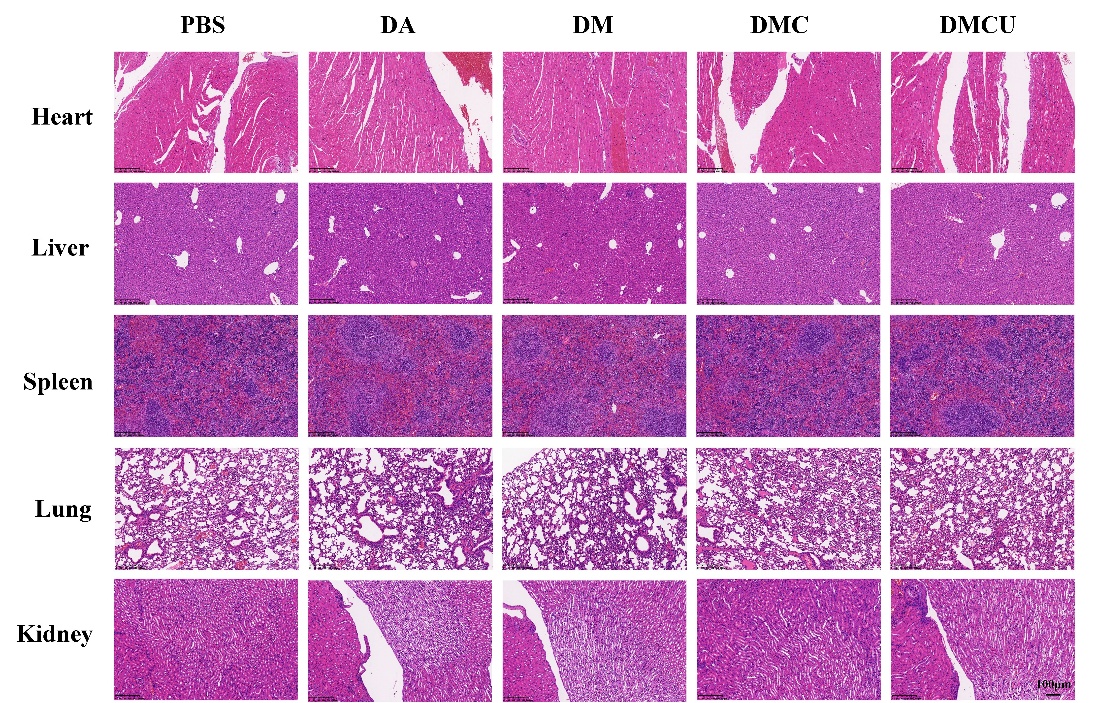


Fig. S14. HE stained sections of heart, liver, spleen, lungs and kidneys of mice treated with different nanomedicine groups.

Fig. S15. Statistical analysis of fluorescence values on day 0 and day 21 after modeling of an orthotopic model of bladder cancer in mice.


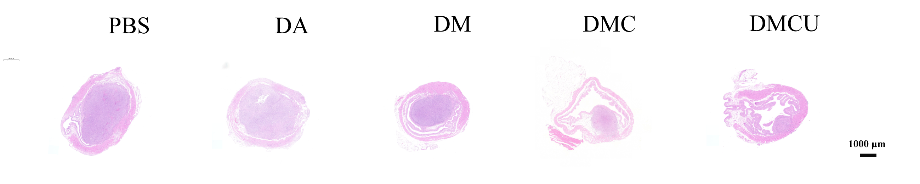


Fig. S16. HE staining analysis of bladders from groups collected on day 21.


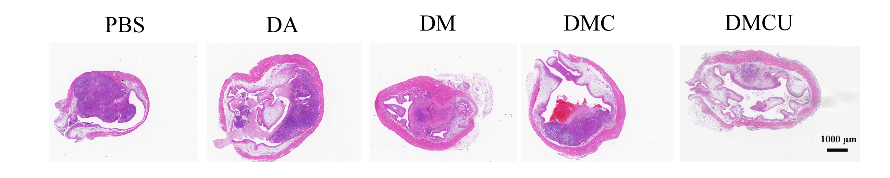


Fig. S17. HE staining analysis of bladders from groups collected on day 14.


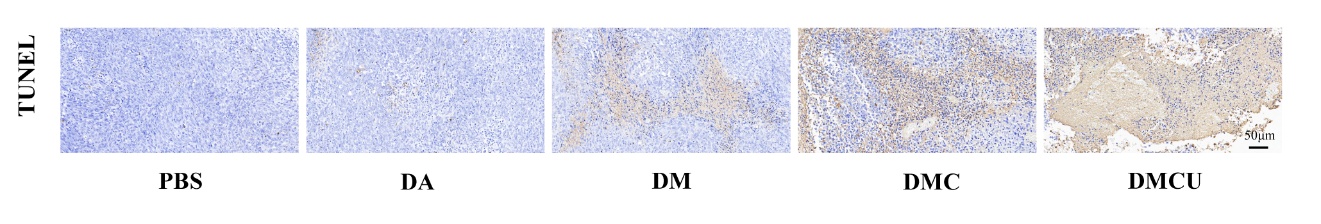


Fig. S18. TUNEL immunohistochemical staining of the bladder of mice treated with different groups of nanomedicines

Fig. S19. Flow cytometric and statistical analysis of TEM activation in the spleens of mice in different treatment groups.

**Movie S1:** Motion performance of DMCU in PBS solution with 0mM urea concentrations.

**Movie S2:** Motion performance of DMCU in PBS solution with 50mM urea concentrations.

**Movie S3:** Motion performance of DMCU in PBS solution with 100mM urea concentrations.

**Movie S4:** Motion performance of DMCU in PBS solution with 200mM urea concentrations.

**Movie S5**: Motion performance of the modified urease (DMCU) in mouse urine.

**Movie S6**: Motion performance of the unmodified urease (DMC) in mouse urine.
